# Longitudinal analysis of the capacities of community health workers mobilized for seasonal malaria chemoprevention in Burkina Faso

**DOI:** 10.1186/s12936-020-03191-y

**Published:** 2020-03-19

**Authors:** Abel Bicaba, Luc Serme, Gaël Chetaille, Gountante Kombate, Alice Bila, Slim Haddad

**Affiliations:** 1 Société d’Études et de Recherches en Santé Publique, Ouagadougou, Burkina Faso; 2 Centre de Recherche du Centre Hospitalier Universitaire de l’Université Laval, Quebec, Canada

**Keywords:** Seasonal malaria chemoprevention, Community health workers’ performance, Training, Evaluation

## Abstract

**Background:**

Seasonal malaria chemoprevention (SMC) relies on community health workers to distribute drugs. This study assessed: (1) the capacity of community-based distributors (CBDs) at the start and end of a campaign and from one campaign to another after training or refresher courses before each round; (2) to what extent CBDs’ experience over several campaigns contributed to measurable increase in their capacities; and (3) to what extent the training and experience of committed CBDs helped the less productive to catch up.

**Methods:**

A longitudinal analysis was conducted in one Burkina Faso health district during the 2017 and 2018 campaigns. A panel including all CBDs was created. Their capacities were observed after: (1) initial training for the 2017 season; (2) refresher training for that year’s fourth round; and (3) initial training for the 2018 season. All were invited to complete a questionnaire at the end of training with 27 multiple-choice questions on their main tasks. Observers noted content coverage and conditions under which training sessions were conducted.

**Results:**

The 612 CBDs showed, on average, high understanding of their tasks from the start of the annual campaigns. Tasks related to communicating with parents and reporting were best mastered. Their capacities grew from round to round and campaign to campaign, after most had undergone training and been supervised by head nurses. The greatest progress was in the technical components, considered more complex, which involved selecting eligible children, choosing the correct drug packet, and referring children to health professionals. Retaining CBDs from one round to the next benefited everyone, whatever their starting level. Groups that initially obtained the lowest scores (women, illiterates, youngest/oldest) progressed the most.

**Conclusion:**

These results confirm the potential of using CBDs under routine programme implementation. Mandating CBDs with targeted tasks is a functional model, as they achieve mastery in this context where investments are made in training and supervision. Losing this specificity by extending CBDs’ mandates beyond SMC could have undesirable consequences. The added value of retaining committed CBDs is high. It is suggested that motivation and commitment be considered in recruitment, and that a supportive climate be created to foster retention.

## Background

As of 2017, 12 Sahelian countries had integrated seasonal malaria chemoprevention (SMC) into their national malaria prevention and control programmes (NMPCP), covering slightly more than half of the 29.3 million eligible children [1]. The implementation of SMC activities tends to follow the World Health Organization (WHO) policy recommendation on SMC for *Plasmodium falciparum* malaria control in highly seasonal transmission areas [2]. Preventive anti-malarial drugs are provided to eligible children during the peak of malaria transmission. The treatment is administered for three successive days and then repeated monthly, for a total of three or four rounds per campaign. The provision of SMC through a door-to-door strategy relies to a large extent on community health workers, called community-based distributors (CBDs). CBDs receive an allowance of approximately US$10 per day. Their mandate typically includes sensitizing the population before each round and administering day-one doses to eligible children. CBDs also have to provide parents with second- and third-day doses and report on their own activity.

There is growing evidence that SMC is effective in real-world conditions [3–5]. These results support the idea that the overall strategy and, more specifically, the involvement of CBDs, is effective and efficient [6]. However, research on CBDs’ performance has been quite limited to date, and little is known about their ability to properly accomplish the whole set of technical, relational, and recording/notification tasks assigned to them. Training and supervision are often identified as major contributors to CBDs’ knowledge, skills, and motivation [7–12]. It has nonetheless been suggested that more evidence is needed about effective approaches to training [8, 10, 12], including in malaria programmes [13].

This paper explores CBDs’ capacities in Burkina Faso. SMC was initiated in that country in 2014. Each campaign targets children from 3 to 59 months of age, and consists of four monthly rounds (July to October). Delivery is mostly door-to-door. SMC was scaled up rapidly and expanded into the 70 health districts of the country in 2019. More than 40,000 (41,183) CBDs were hired and trained to cover 3.6 million targeted children.

This paper presents the results of a longitudinal analysis conducted in one health district in Burkina Faso during the 2017 and 2018 campaigns. First, the study assessed and compared CBDs’ capacities to perform the tasks assigned to them at the beginning and end of a campaign, between the first and fourth rounds, and then from one campaign to the next, at the time of the first round. The second objective was to assess the increase in CBDs’ capacities and the added value of districts’ efforts to retain them and cultivate their commitment. The hypothesis was that the experience acquired by these CBDs would result in a measurable increase in their capacities over time. A final objective was to verify to what extent the successive training sessions and experience acquired by these committed CBDs helped the less productive to catch up with their peers.

## Methods

### Design

The study covered a health district in the country’s central health region. Malaria is meso-endemic and transmission is seasonal; the rainy season lasts 4 to 6 months. A panel was created that included all CBDs in the district. The CBDs’ capacities were observed at three times: (1) in July 2017, after the initial training for the campaign; (2) at the fourth round of the same year, i.e., 3 months later, after the refresher training; and (3) in July 2018, after the annual initial training. The panel was open population-based, including all CBDs in service during one or more of the three observation periods.

### Setting

CBDs were trained all over the country following a cascade model based on WHO [2]. The cascade was initiated by the NMPCP and went through regional and district officers, and then head nurses of primary healthcare centres. All CBDs of the country attended the training, usually provided in primary healthcare centres. A full-day initial training was offered just before the start of the campaign, and then before the second, third, and fourth rounds. Training sessions were standardized based on teaching material provided by the NMPCP. The training included presentations, demonstrations, and simulations covering nine skill sets: (1) being able to justify and explain the reasons for administering SMC; (2) knowing what to do upon arrival in the household; (3) identifying and selecting eligible children and those who should be referred to the health centre; (4) selecting and administering the packet of medicine appropriate to the child’s age; (5) monitoring the child after the medicine is taken and acting appropriately in case of complications; (6) providing parents with second- and third-day doses and showing them the steps they have to take to administer them; (7) instructing parents on how to monitor treated children and what to do if complications arise; (8) providing information on how to prevent malaria and how to obtain care as soon as possible when malaria symptoms appear; and (9) carrying out notification, reporting, and concession-marking activities, i.e., marking compound walls to indicate the household was visited.

### Data collection

Fifty surveyors were trained and then distributed among the various health centres on the day of each training. An observation grid for the training process was used to assess, in the first place, the trainers’ coverage of the content to be delivered (what is SMC; criteria for selecting eligible children; steps of SMC; communication with parents; referral; side effects). A second component of the grid dealt with the conditions in which the training was provided, the interactions between learners and trainers, the dynamism of the trainer, the working environment, and the duration of the session. The grid consisted of 44 items. At each round, two to three observers per site completed the grid and then developed a common grid by consensus.

All CBDs were invited to complete a questionnaire at the end of the training. It included 27 multiple-choice questions, each focused on a specific task. They were asked about their gender, age, education, previous experience as a community health worker, and participation in previous campaigns. The questionnaire was administered face-to-face in the local language; each interview lasted approximately 20 min. The questionnaire was tested and revised beforehand.

### Analyses

Seven scores were constructed to assess the completeness of the training content. Each score corresponded to a group of tasks defined in the training (explanation of the SMC; criteria for selecting children; administration of medication; communication with parents; referral; side effects). Nine intermediate scores and a total score were constructed to assess the CBDs’ capacities in each round. The scores were transposed to a 0–100 scale to facilitate comparisons. All analyses were performed using Stata© 15. Multilevel models adapted to the longitudinal and the hierarchical data structure (each training site being a cluster of CBDs) were used [14]. Regressions were based on censored tobit models when scores were transformed in a 0–100 censored range of values (“metobit” mixed-effects routine in STATA©). The progression of CBDs’ capacities was assessed based on the overall skills score for those who had participated in three rounds. Double difference was used to assess the effects of each round by CBDs’ gender, education, and age.

## Results

The sample consisted of 23 health centres in the first and second rounds; a 24th centre was opened in 2018. No trainer or CBD refused to participate. The number of CBDs varied slightly from one round to another, with head nurses adjusting their staffing based on the CBDs’ workloads and availabilities (panel participation pattern in Additional file 1). About half of the 612 CBDs participated in the three rounds (47%). They included mainly farmers, men, and people with a primary school education who had participated in at least one previous campaign (Table 1). Two-thirds had previously worked as community health workers for other activities.

**Table 1:** Characteristics of CBDs in the first round of the survey (P1-2017)

Characteristics	n	Mean or %
Age	424	32.3
Sex		
Male	305	72.0
Female	119	28.0
Living in the distribution village		
Yes	341	80.4
No	83	19.6
Education level		
No schooling	23	50.4
Literate	69	16.3
Primary school not completed	43	10.1
Primary school completed	289	68.2
Main activity		
Agriculture	284	67.1
Livestock	15	3.6
Commerce	20	4.7
Studies	82	19.4
Other	22	5.2
Years of experience as a CHW		
None	159	37.5
1 year or less	90	21.2
2 years	37	8.7
3 years or more	138	32.6
Number of SMC campaigns in which the CBD has already participated		
None	55	13.0
One	132	31.1
Two or more	237	55.9

Observation showed that the intended contents were covered in the training sessions (Table 2). Content coverage decreased, however, between the first and fourth rounds. Thus, it appeared less attention was given to refresher training, even though the national curriculum directives call for trainers to provide it in the same way. The difference was significant for overall completeness (Δ = − 11.4%; CI_95_ = [− 18.1%:− 4.7%]), SMC explanations (Δ = − 0.30; CI_95_ = [− 0.76:− 0.03]), tasks to be performed at each step (Δ = − 1.74; CI_95_ = [− 2.63:− 0.85]), and communication with parents (Δ = − 0.39; CI_95_ = [− 0.77:− 0.01]). This progression was accompanied by a halving of the duration of the training session (Δ = − 114 mn; CI_95_ = [− 167:− 62]). The comparison of the initial training sessions for the two consecutive campaigns showed no difference in the content actually covered. Finally, the conditions under which the training was conducted varied little from one period to another.

**Table 2:** Completeness of content and conduct of training at each round

Score		Number of items	Round			Variation		Relative variation	
			P1 2017	P4 2017	P1 2018	P1→P4	P1→P1	P1-P4	P1-P1
			(1)	(2)	(3)	(2)-(1)*	(3)-(1)*	(2)-(1)/(1)	(3)-(1)/(1)
Training content									
C1	SMC well explained	6	5.9	5.5	6.0	*−* *0.39*	0.09	*−* *7%*	1%
C2	Selection criteria covered	14	13.2	12.4	13.7	− 0.74	0.52	− 6%	4%
C3	Steps of SMC covered	13	12.2	10.5	12.1	*−* *1.74*	− 0.13	*−* *14%*	− 1%
C4	Communication covered	4	3.9	3.5	3.9	*−* *0.39*	− 0.04	*−* *10%*	− 1%
C5	Referral covered	4	4.0	3.7	4.0	− 0.26	0.00	− 7%	0%
C6	Side effects covered	2	1.8	1.3	1.7	− 0.52	− 0.04	− 29%	− 2%
	Completeness score transformed (range: 0–100)	43	97.7%	86.25%	99.7%	*11.4%*	2.0%		
Conduct of the training									
D1	General conditions	4	2.3	2.2	2.9	− 0.1	*0.6*	− 6%	*24%*
D2	Dynamism of the trainer	3	2.5	2.5	2.7	0.0	0.3	0%	11%
D3	Trainer-participant interactions	5	5.0	4.5	5.0	*−* *0.5*	0.0	*−* *10%*	0%
D4	Work environment	3	1.9	1.7	2.0	− 0.1	0.1	− 7%	7%
	Conduct score transformed (range 0–100)	15	77%	73%	84%	− 3.2%	7.9%		
Duration of the training (min.)									
Mean			229	115	203	*−* *114*	− 27	− 50%	− 12%
Median			195	133	202				
Minimum			24	35	45				
Maximum			470	256	414				

* Crude comparisons, not adjusted. In italics, significant differences between rounds (p < 0.05)

On average, the CBDs demonstrated a high level of understanding of their tasks (Table 3 and Additional file 2). All else being equal, the highest scores were obtained by literate CBDs, those in the intermediate age group (23–39 years), and those who had previously participated in SMC campaigns (Table 4). Previous experience as a community health worker did not seem to be an added value for the SMC programme.

**Table 3:** Mean capacities of CBDs at each of the three observation rounds

Knowledge score		Number of items	Scores by round (%)			Variation*	
			P1 2017	P4 2017	P1 2018	P1- > P4	P1- > P1
			(1)	(2)	(3)	(2)-(1)	(3)-(1)
S1	Reasons for SMC	1	90.3%	96.4%	93.8%	*+ 06.1%*	*+ 03.5%*
S2	What to do upon arrival in the household	3	96%	98%	98%	*+ 01.5%*	*+ 01.6%*
S3	Selection of eligible children	5	82%	87%	85%	*+ 04.4%*	*+ 02.3%*
S4	When to refer the child	3	78%	86%	71%	*+ 07.7%*	*−06.8%*
S5	Selection of drug packet	5	91%	97%	97%	*+ 06.2%*	*+ 05.6%*
S6	Immediate monitoring of child	3	86%	87%	94%	+ 00.7%	*+ 07.8%*
S7	Subsequent monitoring by parents	3	90%	93%	93%	*+ 02.9%*	*+ 03.1%*
S8	Advising parents	3	100%	100%	100%	− 00.1%	− 00.2%
S9	Noting the *concession*	1	96%	98%	88%	+ 02.1%	*−07.7%*
Total	Overall know-how	27	89%	93%	91%	*+ 03.8%*	*+ 02.0%*

* Crude comparisons, not adjusted. In italics, significant differences between rounds (p < 0.05)

**Table 4:** Characteristics of CBDs associated with their know-how

Characteristics of CBDs	Mean score (%)		
	Crude (%)^a^	Adjusted^b^	
		Estimates (%)	Difference (%)^c^
Age			
Q1 (< 23)	89.7	90.2	
Q23 (23–39)	91.6	91.9	*+ 01.6*
Q4 (> 40)	90.4	91.0	+ 00.8
Sex			
Male	91.1	91.5	
Female	90.3	90.6	− 00.9
Education			
Not educated	89.4	89.1	
Educated	91.2	91.8	*+ 02.7*
Experience as CHW			
None	90.8	91.3	
1 year	90.2	91.0	− 00.3
2 years	91.8	91.7	+ 00.5
3 years or more	90.9	91.2	− 00.1
Participation in previous SMC campaigns			
None	89.9	89.6	
1	90.4	91.0	*+ 01.4*
2 or more	91.4	92.0	*+ 02.4*
3 or more	91.1	91.7	*+ 02.1*

^a^Observed values in the sample. Standard errors adjusted for clustering

^b^Predicted values estimated by tobit models. Standard errors adjusted for clustering

^c^Differences in expected outcomes. Significant effects in italics (α = 0.05)

On average, it was in the fourth round that CBDs’ know-how was highest, after the majority had gone through the initial training, been supervised by head nurses in the previous rounds, and attended several refresher courses. The most visible changes from one round to the next had to do with the technical components, considered more complex, which involved explaining the SMC, selecting eligible children, choosing the correct packet of medicine to use (there are two models with different colours depending on the child’s age), and arranging referrals for children with malaria, fever, or complications requiring professional expertise. The CBDs’ average capacities after the initial training for the campaign was higher in the 2018 campaign. However, the gaps noted were slightly smaller than those observed between the beginning and end of the 2017 season. CBDs who had participated in all three rounds of investigation consistently showed higher scores than the others.

Analysis of the panel of CBDs who participated in the three rounds was done to assess the individual progress of perseverant CBDs and compensate for the possible attrition of CBDs who were less motivated or available. Estimates of the progression of the CBDs’ overall skills are presented in Table 5. Between the beginning and end of the 2017 season, estimates suggest an increase of 4.8 points (relative variation of 5.4%). The increase was also significant from one campaign to the next. CBD retention appeared to benefit all groups, whether male or female, better educated or less educated, younger or older. Groups of CBDs who were initially less productive also tended to catch up with their more efficient peers. Women made greater progress than men (+ 6.3 vs. + 4.3), both within a campaign and from one campaign to another (+ 2.8). CBDs with low education levels, who were initially less productive, progressed significantly (+ 5.4 during the 2017 season and 4.6 from one campaign to the next), partially closing the gap with their more educated peers. The youngest and oldest, less productive at the beginning, also progressed more.

**Table 5:** Factors modifying the progression of CBDs’ overall capacities from one round to another (overall score)

	Score by round^a^			Variation^b^			
	P1-2017	P4-2017	P1-2018	Diff. P4-P1	Diff. of diff.	Diff. P1-P1	Diff. of diff.
All	89.2	94.1	92.7	*4.8*		*3.4*	
	[88.1,90.4]	[93.0,95.0]	[91.4,93.9]	*[3.7,5.9]*		*[2.0,4.8]*	
Male (ref.)	89.9	94.2	92.6	*4.3*		*2.7*	
	[88.7,91.1]	[93.0,95.4]	[91.2,94.0]	*[3.1,5.6]*	2.0	*[1.1,4.3]*	*2.8*
Female	87.3	93.6	92.9	*6.3*	[− 0.6,4.5]	*5.5*	*[0.2,5.4]*
	[85.6,89.1]	[91.8,95.5]	[91.1,94.6]	*[4.1,8.5]*		*[3.3,7.8]*	
No schooling (ref.)	86.2	91.7	90.8	*5.4*		*4.64*	
	[84.4,88.1]	[89.8,93.6]	[88.8,92.8]	*[3.1,7.8]*	− 0.8	*[3.3,5.9]*	− 1.5
Educated	90.2	94.9	93.3	*4.5*	[− 3.6,2.0]	*3.05*	[− 4.2,1.3]
	[89.0,91.4]	[93.6,96.1]	[92.0,94.6]	*[2.0,7.1]*		*[1.5,4.6]*	
Q1 age (ref)	87.4	93.7	93.2	*6.3*		*5.8*	
	[85.4,89.4]	[91.7,95.8]	[91.0,95.5]	*[3.8,8.9]*		*[3.0,8.6]*	
Q23 age	90.1	94.5	93.1	*4.4*	− 2.0	*3.0*	− 2.8
	[88.8,91.4]	[93.1,95.8]	[91.7,94.5]	*[2.9,5.9]*	[− 4.9,0.9]	*[1.3,4.7]*	[− 5.9,0.3]
Q3 age	88.8	93.6	91.4	*4.8*	− 1.5	*2.7*	− 3.1
	[87.0,90.6]	[91.7,95.4]	[89.6,93.3]	*[2.6,7.0]*	[− 5.06,2.0]	*[0.26,5.1]*	[− 6.8,0.5]

^a^Predicted values estimated by tobit models. Standard errors adjusted for clustering

^b^Differences in expected outcome and differences in differences. Significant effects in italics (α = 0.05)

## Discussion

The study revealed, first, that CBDs recruited and trained for SMC showed, on average, a high level of understanding of the tasks they had to perform, right from the start of the annual campaigns. They were most proficient at tasks related to communicating with parents or reporting than tasks related to technical components—selecting eligible children, monitoring children after medicine was taken, or referring children for transfer.

These results are nonetheless consistent with another study on community health workers’ performance conducted in Burkina Faso, Nigeria, and Uganda, where 98% of the tasks were mastered by respondents [15]. They are also consistent with those of a recent observational study conducted in the same context with 14 CBDs, which concluded that they “deliver acceptable quality of care” [16]. Studies have also shown that SMC has contributed significantly to reducing both malaria transmission and the biological prevalence of anaemia in children under five [3]. The results of this study, therefore, support the view that malaria prevention benefits from the involvement of community health workers [17].

CBDs are a particular kind of community health worker, as they are recruited periodically, for only 4 months, and have very focused and specific tasks. As such, they are able to learn relatively quickly and comprehensively. This situation contrasts sharply with those of the “generalist” community health workers in Burkina Faso, whose very broad responsibilities have expanded steadily over the years, and who have been shown not to contribute effectively to the treatment of malaria in children [18]. Many authors have, for several decades, stressed the importance of limiting and concentrating the tasks assigned to community health workers and ensuring they have a reasonable and realistic workload [8, 19–22]. There is, therefore, legitimate concern about the decision taken by the national authorities and their partners to extend the CBDs’ mandate to include malnutrition screening as of the second round of the 2018 campaign. This added task was maintained in the CBDs’ activity package in 2019.

Second, the study seems to us to highlight the considerable advantage of fostering commitment among CBDs and retaining them from one campaign to another. On one hand, CBDs who have participated in several campaigns have a better understanding of their tasks. On the other, the capacities of the same CBD increase significantly over the course of a campaign and from one campaign start to the next. The progress is significant even though the baselines are already quite high and margins for progress are naturally limited. Research suggests that the capacities of community health workers tend to wane rather quickly [11, 23, 24] and that refresher training effectively enhances their performance [12, 25, 26]. The CBDs of the 2017 and 2018 campaigns thus seemed to have benefited from the cumulative contribution of each initial campaign training session, the supervision provided by the health teams during each round, and the regular refresher training preceding each round. These repeated exposures helped particularly to remedy their initial weaknesses in the technical spheres, which they had more difficulty grasping.

Third, the retention of CBDs benefited everyone, whether or not those CBDs were initially productive. It was, however, of greater benefit to those who, because of their limited education, age, or gender, had a poorer initial understanding of their tasks. Women completely closed the gap with their male counterparts, and the gaps were narrowing between uneducated CBDs and those with higher levels of education, as well as between the youngest/oldest and the others. This is the first study to examine such changes among community health workers.

Many studies have shown that the personal characteristics of community health workers can be associated with their performance [8, 11, 15, 27], suggesting that the CBDs to be trained should be carefully selected [11, 28]. However, recent reviews reveal that results, particularly with regard to performance compared by type and sex, are not always consistent [12], and that “*studies of workers with lower baseline performance showed greater improvements in prescribing medications…, vaccinating children,… and counseling…*” [29]. As well, the more educated CBDs, who are traditionally more productive, are also less committed and more likely to drop out [8, 11, 15, 27]. The cumulative effect of experience and repeated exposures to training-supervision dyads has reduced or closed the initial gap in understanding, including for the less educated. This raises the question of whether it would not be as, or even more, beneficial to include in the CBD selection process criteria relating to their long-term availability and social acceptability, rather than their learning capacities a priori.

This study appears to us to present the following strengths. It is based on an independent evaluation of an intervention scaled up by national authorities, and not, as is often the case in studies on community health workers, limited to a small-scale experience in selected populations [20]. The fact that the study was conducted on an uncontrolled intervention rather than a pilot or demonstration project reinforces its informative capacity. Using a longitudinal rather than cross-sectional design that covered several rounds and two national campaigns made it possible to evaluate the collective and individual progression of the CBDs’ capacities. The risks of selection bias were contained by selecting a district on the basis of its geographical location rather than the anticipated results of the SMC or the motivation of district leaders, as well as by including all available health centre populations and CBDs in that district. The results regarding CBDs’ capacity increases are robust and statistically significant, even though the margins for progression were a priori limited by the presence of high baseline values. Finally, the internal validity is strengthened by the convergence of the results and their consistency with studies conducted in the country on household and child coverage, and on the impacts of SMC on malaria transmission.

The study also has limitations. It was conducted in only one district, and it is plausible that there would be natural variations in performance between districts and regions of the country. Evidence of CBDs’ good grasp of tasks does not necessarily presume their future performance once in the field, even if it is a prerequisite [30]. Further research would be needed to assess the compliance of CBDs’ practices in the field. Nor does the study provide a precise picture of the specific contributions of the initial training, supervision, or refresher sessions to their progression; rather, it provides more general information on the cumulative effects of these actions. Finally, the limited number of training centres does not provide enough power to explore in detail the associations between CBDs’ capacities and the quality of training by training centre.

## Conclusions

The study provides new knowledge on a recently introduced intervention modality in the health systems of Sahelian countries. In the context of Burkina Faso, it reports on the capacities of the outreach group of community-based distributors, which is its cornerstone. Three lessons emerge from the experience evaluated. First, CBDs master the tasks assigned to them in this context where efforts are regularly invested in training and mentoring community health workers. The model of CBDs mandated for targeted and specific tasks appears to be more functional and appropriate than the ineffective model of generalist community health workers. The loss of this specificity by extending CBDs’ mandates beyond the SMC is potentially hazardous and could jeopardize its effectiveness. Second, CBDs gain greater mastery of the tasks from round to round and campaign to campaign. This progression shows the relevance of a systematic approach to training, supervision, and regular refresher sessions to support routine implementation of the system. Finally, while the increase in CBDs’ capacities relates to all community health workers, it is of particular value to those who, because of their limited education, their age, or gender, have a poorer initial understanding of their tasks.

National authorities should focus their efforts on fostering the retention of recruited CBDs and, in the selection processes, take into account their motivation and long-term commitment, rather than focusing primarily on their presumed learning capacities. They would also benefit from cultivating a climate of trust and a spirit of partnership between the CBDs and the health centres, while also strengthening their credibility among the populations.

## Supplementary information

**Additional file 1.** Participation pattern of the panel.

**Additional file 2.** CBD responses at each round.

## Data Availability

The datasets used and/or analysed in the current study are available from the corresponding author on reasonable request.
